# Molecular mechanisms underpinning sarcomas and implications for current and future therapy

**DOI:** 10.1038/s41392-021-00647-8

**Published:** 2021-06-30

**Authors:** Victoria Damerell, Michael S. Pepper, Sharon Prince

**Affiliations:** 1 Division of Cell Biology, Department of Human Biology, Faculty of Health Sciences, University of Cape Town, Observatory, Cape Town, South Africa; 2 Institute for Cellular and Molecular Medicine, Department of Immunology, SAMRC Extramural Unit for Stem Research and Therapy, Faculty of Health Sciences, University of Pretoria, Pretoria, South Africa

**Keywords:** Sarcoma, Molecular medicine, Drug development

## Abstract

Sarcomas are complex mesenchymal neoplasms with a poor prognosis. Their clinical management is highly challenging due to their heterogeneity and insensitivity to current treatments. Although there have been advances in understanding specific genomic alterations and genetic mutations driving sarcomagenesis, the underlying molecular mechanisms, which are likely to be unique for each sarcoma subtype, are not fully understood. This is in part due to a lack of consensus on the cells of origin, but there is now mounting evidence that they originate from mesenchymal stromal/stem cells (MSCs). To identify novel treatment strategies for sarcomas, research in recent years has adopted a mechanism-based search for molecular markers for targeted therapy which has included recapitulating sarcomagenesis using in vitro and in vivo MSC models. This review provides a comprehensive up to date overview of the molecular mechanisms that underpin sarcomagenesis, the contribution of MSCs to modelling sarcomagenesis in vivo, as well as novel topics such as the role of epithelial-to-mesenchymal-transition (EMT)/mesenchymal-to-epithelial-transition (MET) plasticity, exosomes, and microRNAs in sarcomagenesis. It also reviews current therapeutic options including ongoing pre-clinical and clinical studies for targeted sarcoma therapy and discusses new therapeutic avenues such as targeting recently identified molecular pathways and key transcription factors.

## Introduction

**Fig. 1:**
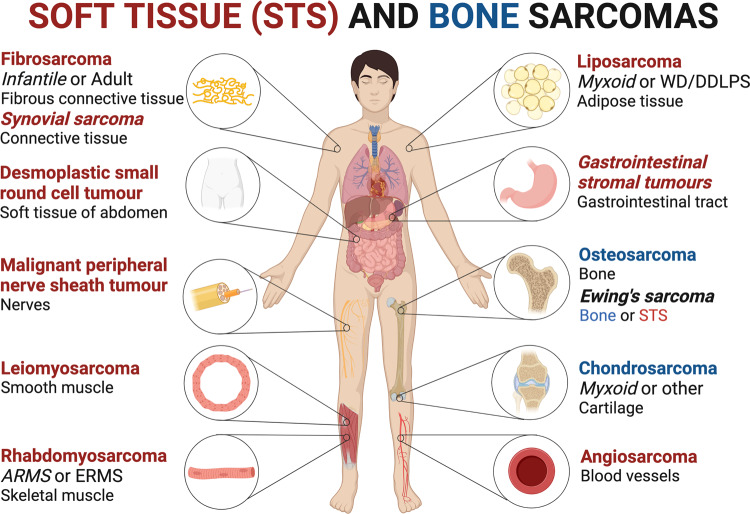
Schematic representation of the most frequently occurring soft tissue (STS) (red) and bone (blue) sarcomas and affected tissues. Sarcomas with a simple karyotype are referred to in italics. ARMS alveolar rhabdomyosarcoma, ERMS embryonal rhabdomyosarcoma, WD/DDLPS well-differentiated/dedifferentiated liposarcoma

Sarcomas are a heterogeneous group of neoplasms derived from tissues of the mesenchyme such as bone, cartilage, muscle, and other connective tissues. Their heterogeneity is highlighted by the identification of over 100 different sarcoma subtypes which vary in pathology, clinical presentation, molecular characteristics, and response to therapy.^1^ Based on histopathological criteria and tissue type of primary manifestation, 80% of sarcomas are categorized as soft tissue sarcomas (STS), 15% as bone sarcomas, and 5% as gastro-intestinal stromal tumors (also known as GISTs) (Fig. 1).^2,3^ While relatively rare, sarcomas are often fatal and because they are of the most aggressive childhood cancers they are responsible for the loss of a significant number of years of life.^4^ Globally, the incidence of STS is around 3–4/100,000 persons per year which accounts for 1% of all adult solid malignant tumors and >20% of all pediatric cancers.^4,5^ The prevalence of sarcomas may however be underestimated since those developing in parenchymatous organs are more often attributed to the organs affected rather than the surrounding connective or supporting tissue.^6^

**Fig. 2:**
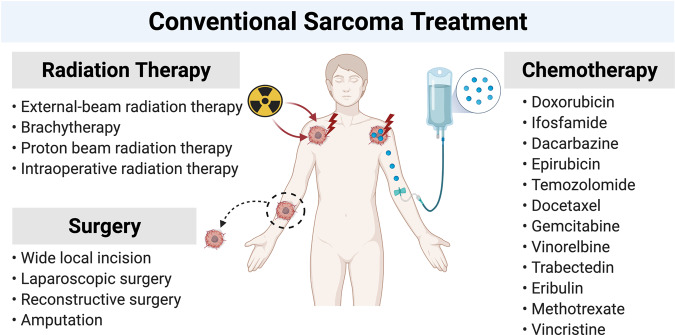
Schematic illustration of conventional sarcoma treatment approaches

The clinical management of sarcomas is highly challenging due to misdiagnosis because they are difficult to differentiate from other malignancies, late diagnosis due to the absence of symptoms, as well as their heterogeneity, aggressive nature, and resistance to current treatment options. Indeed, data published in the National Sarcoma Survey 2020, collected by Sarcoma UK in collaboration with Quality Health, reported that nearly a quarter (23%) of patients had started treatment for another disease before being diagnosed with sarcoma.^7^ Furthermore, Raut et al. reported discordant histopathological diagnoses in up to 25% of sarcomas, of which over half had clinical significance and impact on treatment.^8^ In addition, the absence of symptoms and clinical presentation can result in late referrals to sarcoma specialists which delays diagnosis.^9–11^ Due to the heterogeneity of these tumors, response to conventional treatments such as surgery, radiation, and chemotherapy (Fig. 2) also varies and cannot be translated between different sarcoma subtypes. To date, the only promising curative treatment for localized sarcoma is surgery in combination with pre- or post-operative therapies.^12^ Metastatic sarcomas respond poorly to radiation and chemotherapy which is particularly problematic because one-third of patients develop metastases and about 20% of sarcomas recur.^13^ Furthermore, the 5-year survival rate for localized STS is about 50% and <10% for metastatic STS.^14,15^ While a proposed molecular targeted approach to treatment has gained traction, the molecular mechanisms that drive the sarcoma cells of origin to a transformed phenotype remain to be elucidated. This review, therefore, focuses on the key molecular mechanisms identified to be associated with sarcomagenesis and their potential as novel targets for sarcoma therapy.

## Molecular mechanisms and genomic alterations

Sarcomagenesis is driven by fusion oncoproteins and/or mutations and amplifications that result in activation of oncogenes or loss-of-function of tumor suppressors, leading to unrestrained cell proliferation, invasion, and metastasis. At a genetic level, the karyotype of 15–20% of sarcomas is classified as simple while the remaining is classified as complex. Sarcomas with simple karyotypes are defined by chromosomal translocations which lead to oncogenic fusion proteins which play a central role in their pathogenesis (Table 1).^16^ Sarcomas with complex karyotypes are associated with genetic or chromosomal abnormalities, such as losses, gains, and amplifications, as well as point mutations (Table 2).^17–19^ The rest of this section will review the genetic alterations most frequently associated with simple and complex karyotypes.

**Table 1:** Chromosomal changes observed in a selection of sarcomas with simple karyotype

Type of sarcoma	Chromosomal translocation	Fusion gene	Frequency (%)	Reference
Ewing’s sarcoma (EwS)	﻿t(11;22)(q24;q12)	﻿EWSR1-FLI1	﻿85	^290^
	t(21;22)(q22;q12)	EWSR1-ERG	5–10	^290^
	t(7;22)(q24;q12)	EWSR1-ETV1	<1	^290^
	t(17;22)(q21;q12)	EWSR1-ETV4	<1	^290^
	t(2;22)(q33;q12)	EWSR1-FEV	<1	^290^
﻿Clear cell sarcoma	﻿t(12;22)(q13;q12)	﻿EWSR1-ATF1	﻿>90	^291^
Myxoid liposarcoma (MLP)	t(12;16)(q13;p11)	FUS–CHOP	95	^292–294^
	﻿t(12;22)(q13;q12)	EWSR1-CHOP	5	^295,296^
Extraskeletal myxoid chondrosarcoma	t(9;22)(q22;q12)	EWSR1-NR4A3	62	^297^
	t(9;17)(q22;q11)	TAF2N-NR4A3	27	^297^
	t(9;15)(q22;q21)	TCF12-NR4A3	4	^297^
﻿Desmoplastic small round cell tumors (DSRCT)	t(11;22)(q13;q12)	EWSR1-WT1	≥86.3	^298,299^
Alveolar rhabdomyosarcoma (ARMS)	﻿t(2;13)(q35;q14)	﻿PAX3- ﻿FKHR	55	^300^
	t(1;13)(q36;q14)	PAX7- ﻿FKHR	22	^300^
	t(2;2)(q35;p23)	PAX3-NCOA1	<10	^301,302^
	t(2;8)(q35;q13)	PAX3-NCOA2	<10	^301^
Alveolar soft part sarcoma	﻿t(X;17)(p11;q25)	﻿ASPSCR1-TFE3	100	^303,304^
Synovial sarcoma	﻿t(X;22)(p11.23;q11)	﻿SS18-SSX1	>61	^305,306^
	t(X;18)(p11.21;q11)	SS18-SSX2	<37	^305,306^
	t(X;18)(p11;q11)	SS18-SSX4	Rare	^307^
﻿Infantile fibrosarcoma	﻿t(12;15)(q13;q25)	﻿ETV6-NRTK3	≥87.2	^308–310^

**Table 2:** Frequent genetic alterations observed in sarcomas with complex karyotypes

Type of sarcoma	Genetic alterations	Genes affected	Frequency (%)	Reference
Leiomyosarcoma (LMS)	Deletions	*PTEN*	57–69	^311,312^
		*RB1*	27–59	^311,312^
	Mutations	*TP53*	33–49	^312–314^
		*ATRX*	17–26	^312–314^
		*MED12*	21	^314^
	Amplification	*MYOCD*	70	^315^
Osteosarcoma	Mutations	*TP53*	47–82	^316,317^
		*RB1*	29–47	^316,317^
		*DLG2*	53	^316^
		*ATRX*	29	^316^
	Amplifications	*c-Myc*	39–42	^35,318^
		*CCNE1*	33	^318^
		*RAD21*	38	^318^
		*VEGFA*	23	^318^
		*RUNX2*	Common	^319,320^
Liposarcoma (other than myxoid)	Amplifications	*MDM2*	86–98	^321,322^
		*CDK4*	58–88	^321,322^
		*HMGA2*	75–93	^322^
		*c-JUN*	16–60	^322^
Chondrosarcoma (other than myxoid)	Mutations	*IDH*	50–80	^323,324^
Fibrosarcoma (other than infantile)	Amplifications	*MDM2*	Common	^325^
Embryonal Rhabdomyosarcoma (ERMS)	Deletions	*CDKN2A/B*	23	^326^
	Activating Mutation	*FGR4*	20	^326^
	Activating Mutation	*Ras family*	42	^326^
Angiosarcoma	Mutations	*TP53, PTPRB*	66, 26	^327^
	Overexpression	*VEGF*	80	^327^
Malignant peripheral nerve-sheath tumor (MPNST)	Mutations	*NF1*	87.5	^328^
		*CDKN2A*	75	^328^
		*TP53*	40.3	^328^
		*EED, SUZ12*	Common	^329^
Undifferentiated pleomorphic sarcoma (UPS)	Deletions	*RB1*	30–35	^330,331^

### Alterations in cell cycle regulators

**Fig. 3:**
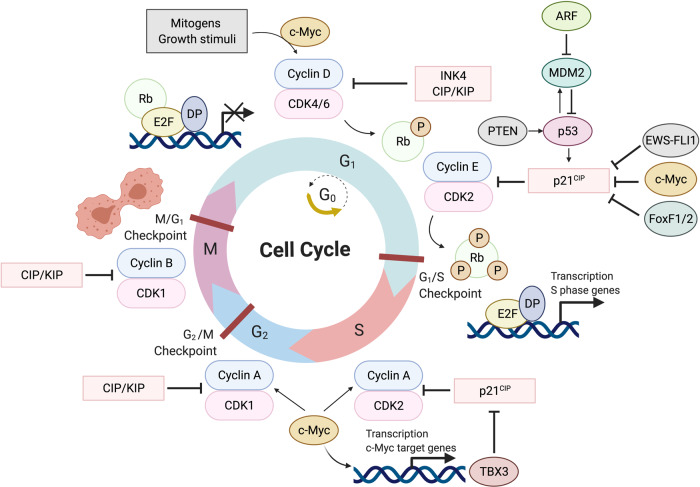
Schematic illustration of the mammalian cell cycle and proteins involved in sarcomagenesis

The mammalian cell cycle is comprised of four distinct phases, namely G_1_ (cells prepare for DNA replication or decide to go into quiescence (G_0_)), S (DNA synthesis), G_2_ (cells prepare for mitosis), and M (mitosis) (Fig. 3). The transition from one phase to another is orchestrated by cyclin-dependent kinases (CDKs) which associate with their regulatory subunits, known as cyclins. When activated, cyclin–CDK complexes phosphorylate substrates that provide the forward impetus through the cell cycle and their inhibition by CDKIs triggers a ‘checkpoint’ that halts the cell cycle.^20^ In mammals there are two classes of CDKIs, CIP/KIP (p21^CIP1^, p27^KIP1^, and p57^KIP2^) and INK4/ARF (p15^INK4b^, p16^INK4a^, p18^INK4c^, and p14^ARF^) which differ in their mechanism of action and specificity.^21^ Alternative splicing of the *CDKN2A* locus gives rise to p16^INK4a^ and p14^ARF^ and p16^INK4a^ blocks G_1_/S transition by interacting with CDK4/6 and inhibiting their association with type D-cyclins.^22^ This impedes CDK4/6-cyclin-D from phosphorylating retinoblastoma (RB) protein, and when hypo-phosphorylated, RB prevents entry into S phase by sequestering E2F transcription factors (TFs) and thereby inhibiting transcription of S phase genes.^23^ Loss of p16^INK4a^ consequently leads to unregulated phosphorylation of RB, activation of E2F and its target genes as well as the transition from G_1_ into S phase. Under conditions of cellular stress, p14^ARF^ sequesters the ubiquitin E3 ligase, mouse double minute 2 homolog (MDM2), that would ordinarily target p53 for proteasomal degradation.^24^ The consequence of this is the stabilization and increase of p53 levels and the transcriptional activation of p53 targets including CDKIs such as p21^CIP1^. This results in cell cycle arrests which can be followed by senescence and/or cell death by for example apoptosis.^25^ The p14^ARF^–MDM2–p53 pathway thus plays a critical tumor suppressor role.

Clinicogenomic profiling of almost 8000 STS patients demonstrated that genetic alterations including loss of DNA copy number and point mutations frequently occur in *RB1* (22%) and *CDKN2A* (22%) with the latter significantly correlating with poor prognosis.^26^ Importantly, loss of the *CDKN2A* locus disrupts the p16^INK4a^-RB and p14^ARF^-p53 tumor suppressor signaling which results in hyperactivation of CDKs and uncontrolled cell cycle progression. Somatic *TP53* mutations, amplifications of *MDM2*, and loss-of-function mutations in *p14*^*ARF*^ have also been observed in a range of sarcomas and are linked with enhanced cell proliferation and survival, metastatic potential, chemotherapy resistance and poor overall patient survival.^26–29^ Furthermore, phosphatase and tensin homolog (PTEN) blocks AKT activation and consequently phosphorylation and translocation of MDM2 to the nucleus, and thus blocks p53 degradation.^30^ A multi-platform profiling of 2539 STS and bone sarcomas revealed loss of PTEN in 38.6% of sarcomas, most commonly in LMS, ARMS, osteosarcoma, chordoma, and epithelioid sarcoma.^31^

The upregulation of positive cell-cycle regulators such as the TFs c-Myc, Forkhead Box F (FoxF1/FoxF2), and T-box transcription factor 3 (TBX3) has also been implicated in sarcomagenesis. Indeed, c-Myc is upregulated in a number of sarcomas including leiomyosarcoma, osteosarcoma, chondrosarcoma, synovial sarcoma, ARMS and EwS.^32–39^ Myc is a basic helix–loop–helix zipper transcription factor that regulates its target genes by binding to a conserved E-box DNA sequence CACGTG.^40^ It mainly exerts its effect on the cell cycle by transcriptionally activating *cyclins* and *CDKs* or by repressing *p15*^*INK4b*^, *p21*^*CIP1*^, and *p27*^*KIP1*^.^41–44^ In rhabdomyosarcoma cells, c-Myc, FoxF1 and FoxF2 are each capable of directly repressing *p21*^*CIP1*^ to promote proliferation and anti-apoptosis.^45,46^ In the case of Ewing’s sarcoma, *p21*^*CIP1*^ is directly repressed by EWS-FLI1 fusion protein.^47^ TBX3 belongs to the developmentally important T-box transcription factor family and is overexpressed in a broad range of sarcoma subtypes which are largely dependent on it for the cancer phenotype.^48^ During S-phase, c-Myc transcriptionally activates *TBX3* in chondrosarcoma and rhabdomyosarcoma cells and TBX3 represses *p21*^*CIP1*^ to confer a proliferative advantage to these cells.^49,50^ A summary of cell cycle proteins involved in sarcomagenesis is illustrated in Fig. 3.

### Alterations in growth factor and pro-survival signaling pathways

**Fig. 4:**
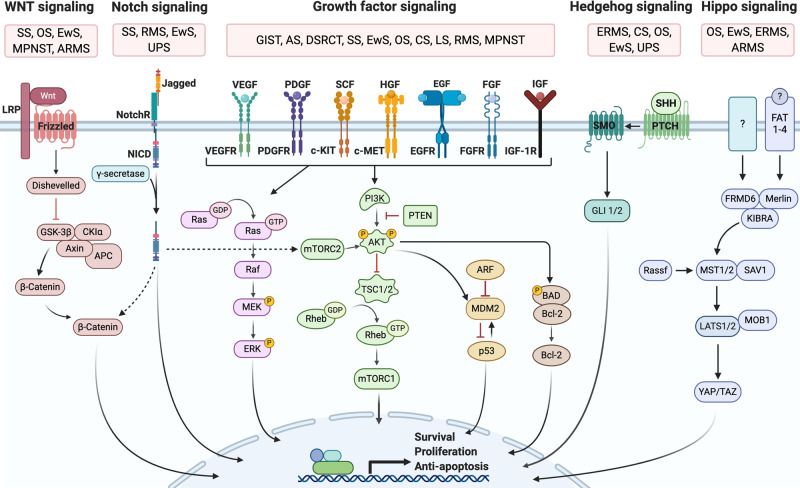
Schematic illustration of key signaling pathways underpinning sarcomagenesis. Wnt signaling: SS,^271^ OS,^272,273^ EwS,^62,274^ MPNST,^275–277^ ARMS,^278^ Notch signaling: SS,^279^ RMS,^279^ EwS,^62^ UPS,^280^; Growth-factor signaling: GIST,^53–55^ DSRCT,^51^ AS,^31,281^ SS,^58^ LS,^31^ CS,^31^ OS,^52^ EwS,^62,282^ RMS,^57,283^ MPNST,^284^; Hedgehog signaling: UPS,^280^ OS,^63^, ERMS,^64^ CS,^65^ EwS,^62^; Hippo signaling: OS,^138,285^ EwS,^286,287^ ERMS,^288^ ARMS.^289^ *Abbreviations*: ARMS alveolar rhabdomyosarcoma, AS angiosarcoma, CS chondrosarcoma, DSRCT desmoplastic small round cell tumors, ERMS embryonal rhabdomyosarcoma, EwS Ewing’s sarcoma, GIST gastro-intestinal stromal tumor, LMS leiomyosarcoma, LS liposarcoma, MPNST malignant peripheral nerve sheath tumor, OS osteosarcoma, SS synovial sarcoma, UPS undifferentiated pleomorphic sarcoma

Most sarcoma subtypes are associated with mutations that result in constitutive activation of pro-survival and growth-factor signaling pathways (Fig. 4). These include the platelet-derived growth factor (PDGF), insulin-like growth factor (IGF), epidermal growth factor (EGF), c-KIT and c-MET pathways which promote tumorigenesis by activating downstream Ras/Raf/MAPK and/or PI3K/PTEN/AKT/mTOR pathways.^31,51–59^ PTEN negatively regulates the PI3K/AKT/mTOR pathway and, as mentioned earlier, is lost in 38.6% of sarcomas leading to the aberrant activation of this pathway.^31,60^ Furthermore, downstream of the PI3K/AKT pathway, the protein kinase mTOR plays a major role in translating proteins for cell-cycle progression, cell growth, and survival, and has therefore become an attractive target for sarcoma therapy.^61^ In addition, TBX3 is an important mediator of rhabdomyosarcomagenesis downstream of the PI3K/PTEN/AKT/mTOR pathway. Indeed, phosphorylation by AKT1 stabilizes TBX3, and TBX3 promotes rhabdomyosarcoma proliferation, anchorage-independent growth and tumor formation.^50^ Furthermore, aberrant stimulation of the WNT, Notch and Hedgehog-GLI signaling pathways promotes proliferation, invasion, and metastasis of a range of sarcoma subtypes.^62–68^ Finally, the Hippo pathway prevents uncontrolled proliferation by phosphorylating and preventing nuclear translocation of the TFs YAP and TAZ, and molecular aberrations within this pathway have been linked to sarcomagenesis.^69^ For example, YAP and TAZ are aberrantly activated in 66% of sarcoma cell lines and 50% of sarcoma patient-derived tissues and this correlated with increased proliferation, anchorage-independent growth and tumor progression.^70^

### Alterations in angiogenic signaling pathways

Angiogenesis which is the formation of new blood vessels, is required for tumor cell growth, invasion and metastasis.^71^ The vascular endothelial growth factor (VEGF) family members VEGF-A, -B, -C, -D and placental growth factor (PGLF) are master regulators of angiogenesis and mediate their biological effects via the surface receptors VEGFR1, VEGFR2, and VEGFR3.^72^ Activation of the VEGF/VEGFR pathway triggers endothelial cell growth and neovascularization from pre-existing vessels; it is therefore not surprising that this pathway is often activated during oncogenesis.^73^ Importantly, upregulation of VEGFs and VEGFRs has been observed in at least 25% of sarcomas, and is linked to advanced tumor stage and poor prognosis.^74–76^ Furthermore, analysis of 115 STS patients revealed significantly higher levels of VEGF tissue concentration in patients with local recurrence and metastasis, which correlated with poor overall survival.^77^ In addition, immunohistochemical analysis revealed that VEGFR1, VEGFR2 and VEGFR3 were expressed at high levels in 61%, 11% and 64% of 275 STS tumors, respectively, and this was significantly associated with higher tumor grade.^78^ Feng et al. also found moderate and high VEGF expression in 37% and 40.7% of synovial sarcoma patients respectively, which was associated with histological grade, cancer staging and metastasis.^75^ Similarly, a correlation between VEGF expression, tumor stage and patient survival has been reported for bone sarcomas. For example, in osteosarcoma patients, those with VEGF-positive tumors had a significantly higher incidence of pulmonary metastases and worse overall survival compared to those with VEGF-negative tumors.^79^ Finally, overexpression of VEGF in STS cell lines led to accelerated growth and formation of highly vascular tumors, pulmonary metastases and chemoresistance in experimental models in vivo.^80^

### Alterations in factors promoting invasion and metastasis

#### Epithelial-to-mesenchymal transition (EMT)/mesenchymal-to-epithelial transition (MET) plasticity in sarcomas

Tumor metastasis involves tumor cells from the primary site invading neighboring tissues, intravasation and transport of tumor cells through the blood or lymphatic systems, and extravasation and tumor growth at secondary sites. In carcinomas, this is facilitated by tumor cells undergoing an EMT which reduces their adhesion properties and enhances their migratory and invasive abilities. Once they reach their destination, they undergo a reverse process termed MET to establish metastases.^81^ EMT is characterized by the downregulation of the epithelial cell–cell adhesion molecule E-cadherin and the upregulation of the TFs Twist-related protein 1 (TWIST-1), Zinc finger E-box-binding homeobox (ZEB)1/2, SLUG, SNAIL, and the EMT inducer transforming growth factor β (TGF-β).^82,83^

**Fig. 5:**
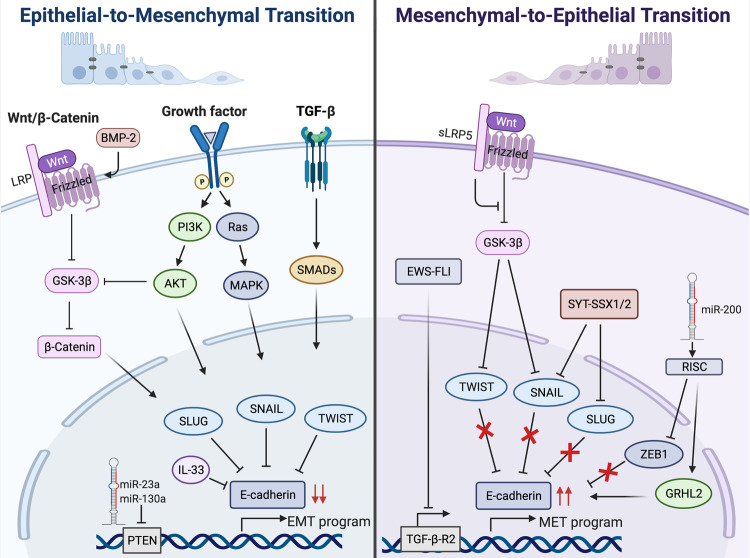
Factors promoting epithelial-to-mesenchymal transition (EMT) and mesenchymal-to-epithelial transition (MET) in sarcomagenesis

Unlike carcinomas, EMT processes in sarcomas are largely unknown and seem paradoxical since they are, by definition, mesenchymal in nature. However, based on recent evidence, Sannino et al. propose that sarcoma cells may reside in a metastable state, and depending on cellular context, can either differentiate towards an epithelial or more mesenchymal phenotype.^84^ This EMT/MET plasticity has been linked to an aggressive phenotype,^84^ and several EMT/MET TFs have been shown to play a role in sarcomagenesis. For example, downstream of the PI3K/AKT/mTOR and MAPK/ERK pathways, SLUG and SNAIL promote EMT-related processes in chondro- and rhabdomyosarcoma cells respectively.^85–87^ On the other hand, downregulation of SNAIL due to an epigenetic switch in chondrosarcoma cells resulted in MET which corresponded with expression of epithelial markers, E-cadherin, maspin, desmocollin 3, and 14-3-3σ.^88^ Similarly, in synovial sarcomas, TGF-β may drive phenotypic switching by upregulating TWIST-1, SNAIL and SLUG which promote cell migration and invasion.^89,90^ The SYT-SSX1 and SYT-SSX2 fusion proteins can reverse the mesenchymal phenotype in synovial sarcoma cells through binding to SNAIL and SLUG respectively, thereby preventing them from repressing *E-cadherin*.^91^ In rhabdomyosarcoma and osteosarcoma cells, miR-200 inhibits *ZEB1* and thereby induces E-cadherin and co-expression of miR-200 and grainyhead‐like transcription factor 2 (GRHL2) results in a multiplicative increase in E-cadherin and morphological changes consistent with MET.^92^ More is known about EMT/MET processes in osteosarcoma cells since several factors including TGF-β, microRNAs (miRs), BMP-2 and Interleukin-33 (IL-33) have been identified to regulate these processes. For example, TGF-β promotes EMT by upregulating SNAIL and subsequently downregulating E-cadherin^93^ and miR-23a and miR-130a induce EMT in osteosarcoma cells by directly downregulating PTEN.^94,95^ In addition, BMP-2 upregulated ZEB2 and activated Wnt/β-catenin signalling in these osteosarcoma cells. This promoted EMT and invasiveness through the inhibition of E-cadherin and increased levels of the mesenchymal markers SNAIL, N-cadherin and vimentin.^96,97^ When the Wnt/β-catenin pathway was blocked with the dominant negative soluble low-density lipoprotein receptor-related protein 5 (sLRP5), EMT was reversed as seen by upregulated levels of E-cadherin and downregulated levels of SLUG, TWIST and matrix metalloproteinases (MMPs).^98^ A recent study demonstrated that IL-33 may promote EMT by downregulating E-cadherin and upregulating MMP-9 and N-cadherin.^99^ Importantly, the upregulation of MMPs does not only occur in osteosarcoma, but has also been reported in several STS where they promote cell invasion and metastasis.^100–102^ Taken together, the above findings indicate that EMT/MET plasticity plays a key role during sarcomagenesis and the factors involved are summarized in Fig. 5.

#### microRNAs

Short non-coding microRNAs have been shown to either inhibit or promote sarcoma metastasis, mainly through the modulation of EMT TFs and MMPs. For example, miR-708-5p, miR-126, and miR-130a impair osteosarcoma EMT, migration, invasion, and metastasis by directly inhibiting *ZEB1* expression and miR-708-5p also downregulates MMP-2, MMP-7 and MMP-9.^103–105^ In a similar manner, overexpression of miR‑30d repressed EwS cell migration and invasion by inhibiting PI3K/AKT/mTOR and MAPK/ERK pathways as well as MMP-2 and MMP-9 levels.^106^ Recently, miR-200b-3p, miR-30c-1-3P, and miR-363-3P were reported to inhibit GIST invasion via direct downregulation of *SNAI2*.^107^ In contrast, miR-182 promotes STS metastasis by downregulating tissue inhibitor of matrix metalloproteinase-1 (TIMP-1) followed by upregulation of its downstream targets and key mediators of cell invasion, MMP-2 and MMP-9.^108^ MicroRNA-135b also upregulated MMP-2 in myxoid liposarcoma which resulted in increased cell invasion in vitro and metastasis in vivo.^109^ Furthermore, miR-181a is overexpressed in high-grade chondrosarcoma and promotes angiogenesis and metastasis by upregulating VEGF and MMP-1.^110^ Taken together, the above studies show that microRNAs are important for sarcoma EMT/MET plasticity and metastasis, and are thus potentially attractive targets for treatment.

#### Extracellular vesicles

Exosomes are extracellular vesicles secreted by several cell types that are important for cell-to-cell communication. Their components include proteins, mRNA, miRNA, and DNA, and have been implicated in the regulation of tumorigenesis and metastasis.^111^ For example, osteosarcoma-derived exosomes were reported to be enriched for proteins implicated in tumor progression, migration, angiogenesis, and metastasis.^112^ Furthermore, gene ontology analysis showed an enrichment for miRNAs associated with tumorigenesis and metastasis in metastatic osteosarcoma-derived exosomes.^112^ In addition, miR-25-3p and miR-92a-3p were shown to be secreted by liposarcoma cells though exosomes and induced interleukin-6 secretion from tumor-associated macrophages, which promoted liposarcoma cell proliferation, invasion, and metastasis.^113^ Since cancer-derived exosomes contribute to metastasis, their disruption may constitute a novel therapeutic strategy.^114^ Indeed, a preclinical study has shown that targeting breast-cancer-derived exosomes with human-specific anti-CD9 or anti-CD63 antibodies significantly reduced metastasis in vivo.^115^ More studies are required to elucidate the exact molecular mechanisms of exosome-related metastasis in sarcomas, and how to target these for treatment.

Exosomes may also represent a useful tool for targeted anti-cancer drug delivery. For example, miR-143 downregulates MMP-13 to suppress osteosarcoma cell invasion and metastasis, and exosome-mediated delivery of miR-143 to osteosarcoma cells significantly reduced their migration.^116,117^ In addition, in a murine sarcoma model, exosome-mediated delivery of siTGF-β1-inhibited TGF-β signaling, tumor growth, and lung metastases.^118^ More investigations are needed to evaluate the potential of exosomes as delivery systems for targeted therapy in sarcomas.

Together, the above sections provide evidence that alterations in several factors and signaling pathways that regulate the cell cycle, angiogenesis, invasion, and metastasis, co-operate to promote sarcomagenesis. Understanding these has been important for modelling sarcomagenesis in MSCs.

## Mesenchymal stem cells as the putative origin of sarcomas

**Fig. 6:**
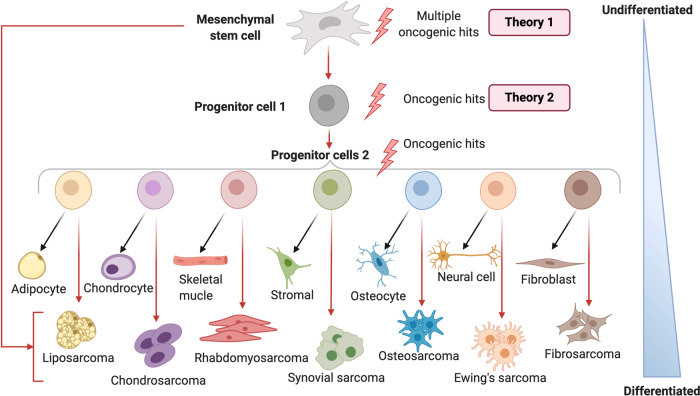
Mesenchymal stromal/stem cell (MSC) differentiation and sarcomagenesis. Schematic representation of malignant transformation of MSCs into several sarcoma subtypes driven by several oncogenic hits (red arrows). During normal development, MSCs mature through different stages (progenitor cells) towards a final differentiated cell such as an adipocyte, chondrocyte, osteocyte, skeletal myocyte, fibroblast, neural, and stromal cell. Theory 1 suggests that oncogenic hits occur in primitive MSCs; theory 2 suggests that oncogenic hits occur in progenitor cells which drives their malignant transformation. The two theories are not mutually exclusive but they feed into a model where sequential genomic alterations in a primitive MSC and/or its progenitor cells result in an accumulation of oncogenic hits followed by malignant transformation

The cells which give rise to sarcomas still remain unclear but recent evidence suggests that MSCs may be sarcoma-initiating cells.^119,120^ MSCs are multipotent stromal/stem cells that are found in most human adult tissues and they give rise to differentiated cell types including adipocytes, chondrocytes, skeletal myoblasts, osteocytes, neural cells and fibroblasts (Fig. 6). Sarcomas are histopathologically classified based on cell-lineage of differentiation and the normal tissue type that they resemble, and two theories have been proposed as to how they arise (Fig. 6). Theory 1 suggests that sarcomas arise from primitive MSCs, which acquire mutations that direct tumorigenesis and theory 2 proposes that progenitor cells acquire mutations at different stages of differentiation which leads to a block in terminal differentiation and subsequent tumor development (Fig. 6).^119^

### Modelling sarcomagenesis using MSCs

Several studies have reported that following the introduction of oncogenic hits, primary MSCs can transform into sarcomas. Indeed, the overexpression of FUS-CHOP combined with loss of p53 in murine MSCs induced liposarcoma-like tumors.^121^ Furthermore, c-Myc overexpression was sufficient to transform murine bone marrow (BM)-MSCs into osteosarcoma in vivo and when combined with loss of the Ink4a/Arf locus, the process was substantially accelerated.^122^ The authors further showed that these osteosarcoma cells consisted of two subpopulations with one showing altered tri-lineage differentiation potential and resistance to conventional anti-cancer drugs. It would however appear that forced expression of a single oncogene in human MSCs (hMSCs) is not sufficient to induce sarcoma development. For example, the expression of EWS-FLI1 alone, was not capable of transforming hMSCs into EwS and expression of FUS-CHOP was only capable of transforming hMSCs into myxoid liposarcoma in the presence of several other oncogenic hits including ﻿p53 and pRB deficiency, hTERT overexpression, c-Myc stabilization, and H-RAS^V12^ mutation.^123,124^ A recent study also showed that overexpression of c-Myc alone enhanced the proliferation of human adipose-derived MSCs (ASCs) and altered their tri-lineage differentiation potential in vitro but it had no effect on their tumor forming ability in vivo.^125^ A combination of c-Myc overexpression and RB knockdown in hMSCs could however transform them into osteosarcoma.^126^ Another study reported that 3H transformed hMSCs (overexpression of hTERT, p53, and pRB degradation) manipulated to overexpress c‐JUN and c‐JUN/c‐FOS developed into fibroblastic and pleomorphic osteoblastic osteosarcomas, respectively.^127^ Combined overexpression of the liposarcoma diagnostic markers, MDM2 and CDK4, increased human 2H transformed BM-MSCs (overexpression of hTERT, p53 degradation) proliferation, migration, and inhibited adipogenic differentiation potential in vitro. However, ﻿ MDM2 and CDK4 overexpression in these MSCs only led to tumor growth in vivo and the formation of dedifferentiated liposarcoma when combined with three additional oncogenic hits (c-Myc stabilization, RB inactivation, and overexpression of H-RAS^V12^).^128^ In contrast to the above findings, Vishnubalaji et al. provided evidence that overexpression of a single oncogene LIN28B in human BM-MSCs resulted in fibromyxoid sarcoma-like tumors in vivo with increased angiogenesis.^129^ The above studies provide overwhelming evidence that at least two oncogenic hits are required to transform hMSCs into sarcomas in vivo.

Despite the evidence from in vitro and in vivo models suggesting that MSCs are the cells of origin of sarcomas, additional studies are necessary to elucidate the mechanisms of MSC transformation into individual sarcoma subtypes.

### Modelling sarcomagenesis using mesenchymal progenitor cells

The possibility that cells of the osteoblastic lineage (pre-osteoblasts, mature osteoblasts, or osteocytes) may be the cells of origin of osteosarcoma has been widely debated.^130–132^ Indeed, p53 is a critical regulator of osteogenesis and studies using conditional and transgenic mouse models showed that inactivation of *TP53* in osteogenic progenitors led to the formation of highly metastatic osteosarcomas which was potentiated by loss of *RB*.^133–136^ Furthermore, constitutive Notch activation in committed murine osteoblasts was sufficient to induce osteosarcoma-like tumors, and when combined with loss of *TP53*, osteosarcoma development was substantially accelerated.^137^ Similarly, upregulation of Hedgehog signaling in p53^+/−^ mutant mice resulted in osteosarcoma development.^138^ Collectively, these studies provide evidence that the loss of p53 is critical for the initiation of osteosarcoma which is consistent with the majority of osteosarcomas exhibiting *TP53* mutations/deletions. Interestingly, Rubio et al. showed that loss of *TP53* and *RB* in osteogenic progenitors derived from murine BM-MSCs, but not ASCs, resulted in the formation of metastatic osteosarcoma.^139^ Additionally, leiomyosarcoma-like tumors were promoted in *TP53* and *RB* null undifferentiated BM-MSCs or ASCs.^139^ Together these observations suggest that not only is a certain level of osteogenic differentiation required for osteosarcoma development but that the source of the cells of the osteogenic lineage is also important. Yang et al. recently provided additional evidence to support this. They showed that consecutive introduction of the oncogenes hTERT, SV40 large T antigen and H-Ras transformed human pre-osteoblasts into osteosarcoma but transformed hMSCs into spindle cell sarcoma.^140^ It is worth noting that osteosarcoma generated from cells of the osteocalcin-lineage i.e. mature osteoblasts, were less osteoblastic compared to osteosarcoma generated from pre-osteoblasts, suggesting that the final differentiation status of osteosarcoma does not necessarily reflect that of their cells of origin.^141^ The final differentiation state of osteosarcomas was proposed to be dependent on silencing of epigenetic regulators such as DNA methyltransferases during osteosarcomagenesis. Furthermore, results from several in vivo studies suggest that osteosarcomas generated from committed progenitor cells are not able to de-differentiate or transdifferentiate into other sarcoma types.^142^ There is also evidence that other sarcomas such as synovial sarcoma, EwS, and myxoid liposarcoma can result from the introduction of the fusion oncoproteins SYT-SSX2, EWS-FLI1, or FUS-CHOP into murine primary mesenchymal progenitors, respectively.^143–146^ Future studies should evaluate whether these oncogenic hits are sufficient to transform human mesenchymal progenitor cells into different sarcoma subtypes.

While the above studies suggest that sarcomas, especially osteosarcomas, can arise from mesenchymal progenitor cells at different stages of differentiation, there is currently a lot more evidence to suggest that sarcomas arise from multiple genetic alterations occurring in primitive MSCs. This may however be due to more studies having been performed with primitive hMSCs and we can therefore not exclude the possibility that sarcomas may arise from either primitive hMSCs or hMSC-derived progenitor cells.

## Molecular targeted therapy

**Fig. 7:**
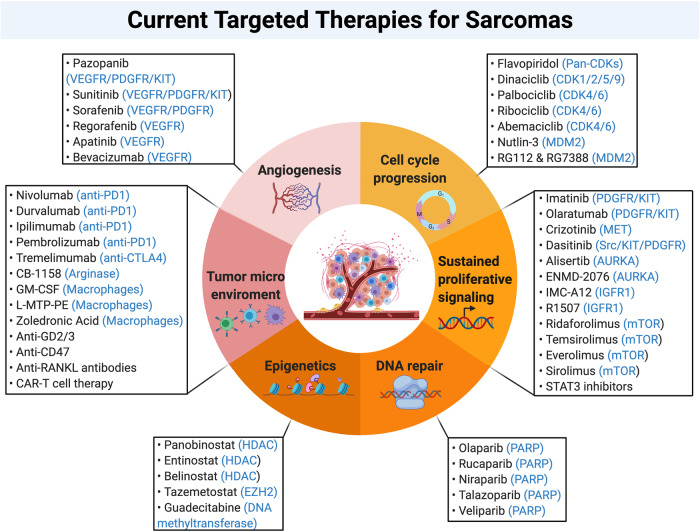
Current targeted therapies for sarcomas. Illustration shows a selection of experimental and approved drugs and their respective targets (highlighted in blue) aimed to inhibit features of sarcomagenesis including cell cycle progression, sustained proliferative signaling, DNA repair, epigenetics, tumor microenvironment, and angiogenesis

The standard treatment for localized sarcomas is surgery combined with neoadjuvant (pre-operative) or adjuvant (post-operative) therapies such as chemotherapy and radiation.^147^ Although patients with localized sarcomas have a high chance of complete recovery with surgery, when their tumors recur or metastasize the prognosis is dismal. This is particularly problematic as 10–20% of sarcomas recur and up to 50% of patients develop metastases.^147,148^ Chemotherapy is the standard form of treatment for metastatic sarcomas; however, the reported median overall survival is only 12 months and <10% of patients have a 5-year overall survival rate.^15,149,150^ There is therefore clearly a need for more effective therapies as the traditional approaches have been mostly ineffective. Targeted therapies may overcome the current therapeutic limitations; however, most sarcoma subtypes have alterations in many signaling pathways and therefore effective therapy will probably need to target a range of pathways. In this section, we review the most relevant pathways that are currently targeted by commercially available drugs, as well as ongoing preclinical and clinical trials on potential novel targeted therapies (summarized in Fig. 7).

## Targeting cell cycle progression

### Cell cycle inhibitors (CDKIs)

CDKIs are being tested for the treatment of sarcomas as they can inhibit aberrant cell cycle activation. First generation CDKIs such as flavopiridol (PubChem CID: 5287969) performed poorly in patients and were associated with toxicity, which is partly due to their lack of specificity.^151^ Second generation inhibitors such as dinaciclib (PubChem CID: 46926350) had more specificity for fewer CDKIs and displayed less toxicity; however, their performance was disappointing in early phase clinical trials.^151^ The major problem with non-selective CDKIs is their inability to differentiate between normal and cancer cells. Therefore, efforts have focused on developing single CDK-specific inhibitors that exhibit maximum anti-tumor effects with minimal toxic side effects. Currently, the most promising agents include the CDK4/6 inhibitors palbociclib (PubChem CID: 5330286), ribociclib (PubChem CID: 44631912), and abemaciclib (PubChem CID: 46220502). For example, CDK4 amplification is typical in over 90% of well-differentiated/dedifferentiated liposarcomas (WD/DDLPS), and palbociclib has demonstrated anti-tumor potential in preclinical studies and a phase II clinical trial of patients with advanced or metastatic WD/DDLPS and resulted in a more favorable progression-free survival (NCT01209598).^151^ Palbociclib may also be beneficial for osteosarcoma treatment since they frequently have disruptions in the pRB pathway, such as loss of p16^INK4a^ and/or amplification of CDK4/6.^152^ Despite the promising preclinical and clinical data, limitations such as acquired drug resistance to CDK4/6 inhibitors are coming to light.^153–155^ In an attempt to overcome these challenges with CDK-monotherapy, ongoing clinical trials are mostly focusing on combination therapies (NCT04129151, NCT03709680, NCT02897375, NCT02784795, NCT03009201, NCT03114527, NCT02343172).

The ubiquitin E3 ligase MDM2 which is responsible for ubiquitinating and targeting p53 for degradation, is often amplified in sarcomas.^156^ This has prompted the development of MDM2 therapeutic inhibitors including nutlin-3 (PubChem CID: 216345) and RG7112 (PubChem CID: 57406853).^25^ Nutlin-3 was shown to repress tumor formation by inducing apoptosis in osteosarcoma xenografts by stimulating the p53 signaling pathway.^157^ Furthermore, RG7112 significantly reduced tumor growth in patients with MDM2-amplified liposarcoma in a phase I clinical trial.^149^ Liposarcomas frequently harbor amplifications of both MDM2 and CDK4, and therefore a combination therapy targeting both factors may have a synergistic effect and lead to a better treatment outcome. Indeed, a preclinical study by Laroche-Clary et al. found that compared to monotherapy, a combination of the MDM2 inhibitor RG7388 (PubChem CID: 53358942) with the CDK4/6 inhibitor palbociclib led to significantly reduced tumor growth in DDLPS xenografts and increased progression-free survival.^158^

## Targeting growth receptors and pro-survival signaling molecules

### Tyrosine kinase inhibitors (TKIs)

TKIs represent a highly successful form of targeted therapy for sarcomas. For example, the c-KIT, PDGF, and VEGF receptors are currently being targeted in approved therapeutics. Eighty percent of GISTs harbor mutations in *c-KIT* and 10% harbor mutations in *PDGFRα*; this leads to constitutive ligand-independent activation of these receptors which promotes uncontrolled cell proliferation and anti-apoptosis.^159^ Molecular-targeted therapy has significantly improved the prognosis of GISTs which are intrinsically resistant to chemotherapy and radiation. Indeed, one of the first FDA-approved molecular targeted drugs is imatinib (PubChem CID: 5291), a TKI which inhibits both c-KIT and PDGFR.^160^ Imatinib has shown great efficacy for the treatment of GISTs and is currently used as the first line treatment for these tumors.^161^ A study on GISTs in the United States by Demetri et al. reported a **>**50% response to imatinib in which the estimated 1-year overall survival was 88%.^162^ Long-term data confirmed the success of imatinib with a 5-year overall survival of about 50%.^163^ Based on these findings, the therapeutic use of imatinib has been extended to other sarcomas that contain *c-KIT* and *PDGFR* mutations. However, the response rates of other sarcomas have mostly been poor.^164^ Indeed, a phase II clinical trial which assessed the performance of imatinib on 22 metastatic or relapsed KIT- or PDGFR-positive sarcomas only showed partial response in a single patient.^165^ Therefore, there was no correlation between the response to imatinib and expression levels of PDGFR/KIT.

Pazopanib (PubChem CID: 10113978) is another TKI that targets VEGFR with high affinity and both c-KIT and PDGFR with lower affinity.^166^ Results from the PALETTE phase III clinical trial showed that pazopanib was beneficial in treatment-resistant metastatic sarcomas and improved progression-free survival by 3 months.^167^ While this led to its FDA-approval as a second-line treatment for advanced STS, treatment with pazopanib has been linked to side effects such as diarrhea, weight loss, nausea, fatigue, and hypertension.^167^ A recent phase II clinical trial (GISG-04/NOPASS) by the German Interdisciplinary Sarcoma Group assessed the effect of pazopanib as preoperative therapy in 21 STS patients; beneficial effects were only observed in a single patient.^168^

Sorafenib (PubChem CID: 216239) is a multi-TKI that inhibits VEGFR and PDGFR and results from phase II clinical trials indicated that it had moderate activity as a second-line therapy for metastatic STS.^169–171^ Furthermore, for patients with desmoid tumors, sorafenib significantly prolonged progression-free survival.^172^ A recent phase II clinical trial showed that a combination of sorafenib with the cytotoxic agent ifosfamide (PubChem CID: 3690) achieved significant clinical benefit in advanced STS patients.^173^ Another phase II clinical trial used sorafenib in combination with the cytotoxic agent dacarbazine (PubChem CID: 135398738) in leiomyosarcoma, synovial sarcoma, and MPNST patients; modest activity and a favorable disease-control rate were observed, although the combination also increased the potential for significant toxic side effects.^174^ Importantly, a pooled analysis of several clinical trials between 2009 and 2016, showed that treatment with pazopanib, sorafenib and sunitinib (PubChem CID: 5329102) was linked to significantly increased risk of toxicity and severe adverse side effects.^175^

Small molecule inhibitors that target TKs may be associated with less adverse side effects. For example, the small molecule inhibitor ZD6474 (PubChem CID: 3081361) targets TKs including VEGFR-2 and EGFR, and in preclinical osteosarcoma studies was shown to block cell proliferation and enhance cell cycle arrest and cell death by suppressing the PI3K/AKT and MAPK/ERK pathways.^176^ Furthermore, the combination of ZD6474 with the COX-2 inhibitor celecoxib (PubChem CID: 2662) resulted in an additive or synergistic anti-tumor effect in vitro and in vivo.^176^ To date, ZD6474 has not yet being tested in clinical trials for sarcoma treatment, but it significantly improved the progression-free survival for medullary thyroid cancer in a phase II clinical trial.^177^

Although the use of TKIs results in clinical efficacy in STS other than GISTs, one of the biggest challenges is the lack of validated predictive biomarkers for patients who are most likely to respond positively to TKI treatment.^178^ Furthermore, TKIs still have limited therapeutic application due to their side effects such as hypertension, arterial and venous thromboembolic events, and hand-foot skin reactions.^179,180^

### IGFR inhibitors

Monoclonal antibodies that target IGFR-1, such as cixutumumab (IMC-A12) or R1507, showed modest clinical benefit for EwS, liposarcoma, osteosarcoma, rhabdomyosarcoma, and synovial sarcoma patients in phase II clinical trials.^181,182^ However, most patients who initially responded to therapy developed drug resistance and suffered from disease recurrence.^183^ Therefore, combination therapies are currently under investigation in many preclinical studies. For instance, a combination of inhibitors to IGFR and CDK4/6 profoundly repressed the PI3K/mTOR pathway and had a synergistic anti-tumor effect in vitro and in vivo in EwS.^184^

### mTOR inhibitors

The mTOR inhibitor ridaforolimus (PubChem CID: 11520894), an analogue of rapamycin, has shown promising results for the treatment of several sarcomas. Results from a phase II clinical trial showed that ridaforolimus had clinical benefit and only mild to moderate adverse side effects for patients with metastatic or unresectable STS and bone sarcomas.^185^ In contrast, an international randomized phase III clinical trial demonstrated that second-line treatment with ridaforolimus delayed sarcoma progression to only a small extent.^186^ Furthermore, the placebo and ridaforolimus-treated groups had comparable overall survival rate and therefore the FDA rejected the approval application for ridaforolimus in 2012. Other inhibitors of mTOR namely everolimus (PubChem CID: 6442177), temsirolimus (PubChem CID: 6918289) and sirolimus (PubChem CID: 46835353) were assessed in single-agent clinical trials; however, results were mostly disappointing.^149^ Preclinical phase investigations are therefore currently under way for combination therapies. For example, co-treatment with ridaforolimus and palbociclib resulted in a synergistic anti-tumor effect in a range of sarcoma cell lines and in a murine fibrosarcoma model.^187^

## Other therapeutic avenues

### Inhibition of epigenetic regulators

Histone deacetylase inhibitors (HDACi) are powerful epigenetic regulators that affect tumor cells by interfering with cell growth, inducing apoptosis and inhibiting angiogenesis.^188^ Although preclinical studies have shown promising anti-cancer activity of the HDACi panobinostat (PubChem CID: 6918837), monotherapy in advanced STS did not show clinical benefit in phase II clinical trials; different combination therapies are therefore currently in development.^189,190^ For example, HDAC inhibitors enhanced the anti-cancer effect of pazopanib against sarcoma cells, and this effect was even more pronounced in combination with the TK inhibitor, neratinib.^191,192^ Furthermore, results from a phase I clinical trial found panobinostat to increase the efficacy of the topoisomerase II inhibitor epirubicin (PubChem CID: 41867) and this led to clinical benefit and has the potential to reverse anthracycline resistance.^193^ Interestingly, tazemetostat (PubChem CID: 66558664), a lysine methyltransferase inhibitor of the histone modification enzyme enhancer of zeste homolog 2 (EZH2), was approved by the FDA in 2020 as the first epigenetic therapy for solid tumors and is used to treat advanced or metastatic epithelioid sarcoma.^194^ EZH2 is upregulated in numerous sarcomas including synovial sarcoma, rhabdomyosarcoma, EwS, and MPNST, where it promotes tumorigenesis and cancer progression.^195^ Importantly, the inhibition of EZH2 in these sarcomas resulted in cell death and a reduction in tumor growth. Thus, EZH2 represents a potential therapeutic target in a range of sarcomas, and clinical studies should therefore investigate the effect of tazemetostat in other sarcoma subtypes. The treatment of ARMS and ERMS with the DNA methyltransferase inhibitor guadecitabine (PubChem CID: 135564655) also reduced cell growth, induced apoptosis and differentiation, and repressed ARMS tumor growth in vivo; this occurred by activating canonical Hippo signaling and downregulating YAP1, a known tumor promoter of RMS.^196^

### Poly(ADP-ribose) polymerase (PARP) inhibitors

PARP enzymes are important players in the repair of DNA single-strand breaks through the base-excision repair pathway, and their inhibition was found to potentiate the cytotoxic effect of DNA-damaging agents.^197,198^ Inhibitors of PARP (PARPi) represent a novel class of anti-cancer agents that are especially effective against cancers with DNA-repair defects where they induce synthetic lethality.^199^ Recently, PARP inhibitors have also been identified as promising agents for sarcoma treatment.^200^ Olaparib (PubChem CID: 23725625), an FDA-approved PARPi, is currently in Pediatric MATCH phase II trials for advanced, recurrent, and refractory STS and bone sarcomas resulting from defects in DNA damage repair genes (NCT03233204 and NCT03155620). Furthermore, multiple clinical trials are either underway or have investigated the performance of chemotherapy–PARPi combinations (NCT02044120, NCT01858168, NCT02116777, NCT03880019). Indeed, a Phase Ib clinical trial assessed the combination of olaparib and the DNA alkylating agent trabectedin (PubChem CID: 108150) which are both known to cause accumulation of single-strand and double-strand DNA breaks. Results showed manageable toxicity of the combination and encouraging anti-tumor activity in advanced STS and bone sarcoma patients (NCT02398058).^201,202^ Perez et al. recently reported that a synergistic effect can be achieved when olaparib is combined with the DNA damaging agent doxorubicin (PubChem CID: 31703) in vitro and in sarcoma patient-derived xenograft (PDX) models.^203^ The authors further showed that this combination was most effective in tumors that expressed high levels of pH2AX and MAP17. Furthermore, due to the radiosensitizing activities of PARPi, a phase Ib study is currently investigating the effects of combining olaparib with concomitant radiotherapy to treat locally advanced/unresectable STS (NCT02787642). Taken together, PARPi in combination with DNA damaging agents/radiotherapy may be an effective treatment strategy for both STS and bone sarcomas.

### Immunotherapy

The most popular immunotargets include programmed cell death protein (PD)-1 and its ligand PD-L1 as well as cytotoxic T-lymphocyte-associated protein (CTLA)-4.^204^ Clinical trials are currently investigating the potential of monoclonal antibodies (mAB) against PD-L1 and CTLA-4 to treat DDLPS and pleiomorphic liposarcoma (NCT02500797 and NCT03114527). Other ongoing phase II clinical trials are investigating the effect of the PD-L1 inhibitor durvalumab in combination with pazopanib or tremelimumab (CTLA-4 inhibitor) to treat advanced STS (NCT03798106 and NCT02815995). However, pooled analysis of results from recent phase II clinical trials revealed that, as single agents or in combination therapy, PD-1/PD-L1 antagonists have limited activity in unselected STS.^205^ Whereas patients with undifferentiated pleomorphic sarcoma and alveolar soft part sarcoma showed the highest overall response and non-progression rate, leiomyosarcoma patients showed the lowest overall response and non-progression rate. This suggests that the success of anti-PD1/PD-L1 treatment is largely dependent on specific sarcoma subtypes. Molgora et al. further demonstrated that the inhibition of TREM2, a pro-tumorigenic marker of tumor-associated macrophages, with an anti-TREM2 mAB, substantially increased the performance of anti-PD-1 treatment in a sarcoma mouse model.^206^ This combination strategy is of particular interest since it effectively targets the immunosuppressive tumor microenvironment and enhances anti-tumor immune responses. The efficacy of monotherapy with a CTLA-4 inhibitor has only been evaluated in one pilot study using ipilimumab; however, no clinical benefit was observed in patients with synovial sarcoma.^207^ Recently, the IMMUNOSARC study, a phase II clinical trial which investigated the potential of treating advanced STS with a combination of the multi-targeted TKI sunitinib with the PD-1 inhibitor nivolumab, was completed. Promising results were observed with an overall and progression free survival of 77% and 50%, respectively, at 6 months (NCT03277924).^208^ Chimeric antigen receptor T (CAR-T) adaptive cell therapy involves the isolation of a patient’s own T-cells and modifying them to express a CAR that recognizes a specific tumor antigen and then reinjecting them into the patient.^209^ Recognition of the tumor cells by the CAR activates T-cell proliferation and elimination of the tumor cells. CAR-T cell therapy has proven to be promising for hematological cancers and is currently under investigation for solid tumors including sarcomas.^210–212^ A phase I/II clinical trial showed that HER2-CAR-T cells travel to the site of human epidermal growth factor receptor 2 (HER2)-positive sarcomas and persist for more than 6 weeks; the median overall survival ranged from 5.1 to 29.1 months without inducing toxicity (NCT00902044).^213^ A CAR-T cell therapy pilot study is currently undergo where the T-cells from myxoid liposarcoma patients were genetically engineered to recognize NY-ESO1, an antigen expressed in 80–90% of myxoid liposarcoma patients (NCT02992743).^214^ Interestingly, 70–80% of synovial sarcomas also express NY-ESO1, and T-cell receptor (TCR) treatment has recently gained FDA approval for patients with HLA-A*201, HLA-A*205, or HLA-A*206 allele-positive advanced synovial sarcoma.^215–217^ It may therefore be worthwhile to evaluate whether CAR-T cell therapy is superior to TCR treatment in synovial sarcomas. A phase I clinical trial that utilizes CAR-T cell therapy to target EGFR and CD19 to treat children and young adults with recurrent/refractory solid tumors is ongoing (NCT03618381). Preclinical studies have proven that CAR-T cell therapy targeting sarcoma-associated antigens is effective and ongoing clinical trials are evaluating its therapeutic potential (reviewed in ref. ^218^).

## Future directions

### Targeting oncogenic TFs

Historically, TFs were considered undruggable, but there is increasing preclinical and clinical evidence that their activity can be targeted. Indeed, TFs can be targeted by inhibiting their interactions with DNA or protein co-factors or by decreasing their protein stability through targeting the proteasome.^219,220^ Aberrant TF activity plays a critical role in simple and complex karyotype sarcomas, and recently there have been advances to target oncogenic fusion TFs in sarcomas. For example, trabectedin is clinically effective against leiomyosarcomas and liposarcomas where it interferes with the ability of FUS-CHOP to bind its target promoters.^221–223^ Trabectedin was FDA-approved for these sarcoma subtypes in 2015 and is currently in clinical trials for other sarcoma subtypes (NCT02367924, NCT02275286, NCT04076579, NCT01303094, NCT04067115).

Emerging evidence has revealed that genome-editing systems and genetic approaches including clustered regularly interspaced short palindromic repeats associated protein 9 (CRISPR-Cas9) and RNA interference (RNAi) have therapeutic potential by directly targeting fusion oncogenes or their respective DNA-binding motifs. Indeed, CRISPR-Cas9-mediated knockdown of PAX3-FOXO1 significantly reduced colony formation in a human myoblast model.^224^ Knockdown of *EWSR1-FLI1* with CD99-targeted nanoparticles carrying Cas9-EWSR1 sgRNA RNP led to reduced tumor growth in EwS xenografts.^225^ Deletion of the GGAA-microsatellite sequence regulating the activation of NR0B1 by *EWSR1/FLI1* using CRISPR-Cas9 led to reduced EwS cell proliferation and anchorage-independent growth.^226^ GGAA-microsatellite repeats were found to be specifically active only in EwS, and silencing multiple repeats using CRISPR-Cas9 strongly decreased the expression of putative EWS-FLI1 target genes.^227,228^ Furthermore, targeting of a single SOX2-regulating GGAA-microsatellite enhancer abrogated EwS tumor growth in vivo.^228^ Martinez-Lage et al. recently proposed that non-homologous end joining (NHEJ) CRISPR-mediated deletion of fusion oncogenes is an efficient and selective strategy for cancer cell elimination. The authors showed that CRISPR-mediated *EWR1-FLI1* deletion inhibited tumor growth in EwS xenografts and PDX models. Moreover, a combination of *EWSR1-FLI1* deletion and doxorubicin was more effective compared to either monotherapy alone in xenograft models.^229^ Inhibiting expression of EWS-FLI1,^230–232^ PAX3-FOXO1,^233,234^ and SYT-SSX^235,236^ by RNAi in vitro also significantly reduced cell viability and induced cell death. However, these anti-tumor effects were not always confirmed in vivo and siRNA delivery using nanoparticles, liposomes and recombinant exosomes has had limited success.

Approaches to target fusion oncoproteins by proteasome degradation or post-translational modifications have also been investigated. For example, treatment of synovial sarcoma cells with the HDACi FK228 (PubChem CID: 5352062) led to degradation of SYT-SSX and decreased cell viability and induced apoptosis.^237^ Similarly, treatment of EwS cells with entinostat (PubChem CID: 4261), a HDAC1/3 inhibitor, led to reduced EWS-FLI1 expression, a G_0_/G_1_ cell cycle arrest, DNA damage, caspase activation and apoptosis.^238^ PLK1, a serine/threonine kinase, phosphorylates and stabilizes PAX3-FOXO1 in ARMS cells, and when it was inhibited with a small molecule inhibitor BI 2536 (PubChem CID: 11364421), PAX3-FOXO1 was ubiquitinated and degraded and this corresponded with tumor regression in a xenograft mouse model.^239^ Similarly, 100% tumor regression was obtained in xenograft mouse models of ARMS using the PLK1-inhibitor volasertib (BI 6727) (PubChem CID: 10461508).^240^ Furthermore, PAX3-FOXO1 activity is dependent on the epigenetic regulator bromodomain-containing protein 4 (BRD4) and inhibition of BRD4 with the small molecule inhibitor JQ1 (PubChem CID: 46907787) resulted in anti-cancer activity in preclinical models.^241^ The advantages and drawbacks of targeting oncogenic fusion TFs are further discussed in a recent editorial.^242^

The oncogenic c-Myc, which is amplified in a variety of sarcomas with simple and complex karyotypes, has been successfully targeted in preclinical studies and clinical trials using multiple approaches. Omomyc, a dominant negative c-Myc, interferes with the ability of c-Myc/Max complexes to bind and activate their target genes.^243^ In a preclinical model of lung adenocarcinoma, Omomyc slowed tumor growth, and when combined with paclitaxel (PubChem CID: 36314), almost completely abrogated tumor growth.^244^ Due to its high anti-cancer potential and limited side effects, Omomyc is about to reach clinical trials for lung, breast and colorectal cancers. Future preclinical and clinical studies should investigate the potential of Omomyc to treat c-Myc-driven sarcomas. BRD4 is involved in regulating c-Myc transcription, and its inhibition using JQ1 resulted in anti-cancer activity in rhabdomyosarcoma and EwS, among other cancers.^241,245–248^ While JQ1 treatment significantly reduced EwS cell proliferation and tumor growth in vivo, it did not result in a downregulation of c-Myc, indicating that BRD4 does not target c-Myc in EwS.^249^ Proteolysis targeting chimaeras (PROTACs), a technology that utilizes the ubiquitin–protease system to target proteins for proteasomal degradation, has shown better success than JQ1 in targeting BRD4.^250^ For example, ARV-825 (PubChem CID: 92044400) was designed to target BRD4 and it has greater ability than JQ1 or other small-molecule BRD4 inhibitors to downregulate levels of c-Myc and its downstream target genes.^251,252^ ARV-825 and ARV-771 (PubChem CID: 126619980) also have anti-cancer potential in MPNST cells by downregulating BRD4, inhibiting cell viability and inducing apoptosis.^253^ The PROTAC BET-d260 significantly downregulated BRD2,3,4 and c-Myc in a number of osteosarcoma cell lines and this was associated with tumor growth inhibition and apoptosis.^254^ Furthermore, PROTAC MD-224 (PubChem CID: 131986956) which efficiently targets MDM2, induced complete and durable tumor regression in leukemia cells in vivo, and it would be worth evaluating its activity in MDM2-driven sarcomas such as liposarcomas.^255^ Future clinical trials should evaluate whether targeting TFs either alone or in combination with other targeted therapeutic approaches can lead to more effective therapies and better outcomes for sarcoma patients.

### Personalized precision medicine

**Fig. 8:**
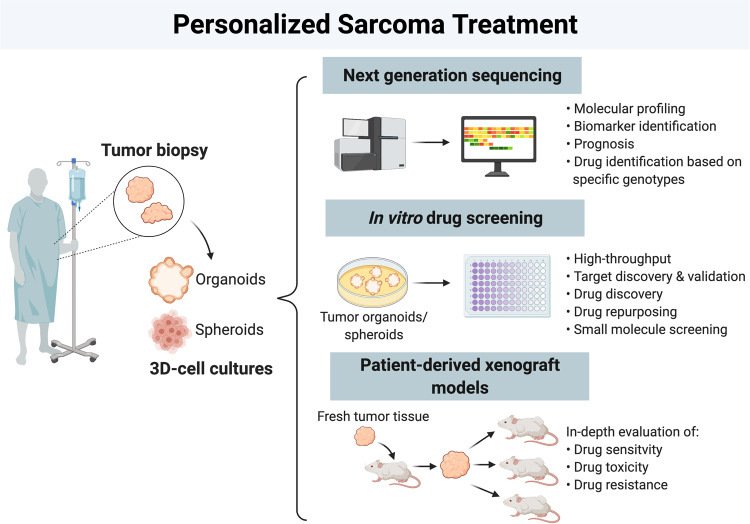
Schematic illustration of personalized sarcoma treatment depicting workflow from sarcoma biopsy to next generation sequencing, in vitro drug screening, and patient-derived xenograft (PDX) models

Next generation sequencing (NGS) tools allows for rapid and cost-effective sequencing of whole genome DNA and transcriptomic RNA profiles which can be used for either diagnostic or therapeutic purposes. So far, they have enabled the generation of large gene expression signatures of downstream targets of genetic or chromosomal aberrations in specific sarcoma subtypes. These NGS tools are redefining the way we diagnose and treat sarcomas, as sarcomas are difficult to fully characterize even by expert pathologists. Rationally chosen drug treatment based on NGS data in individual patients could provide clinical benefit and should become the norm for sarcoma diagnosis and treatment. Furthermore, 3D-cell culture models such as spheroids and organoids derived from tumor tissue from a patient represent novel tools that can be used to identify personalized drug treatments. Importantly, these models are able to overcome limitations associated with traditional 2D-monolayer cell cultures and more realistically reflect tumor heterogeneity, cell-extracellular matrix interactions and tumor microenvironment.^256^ Similarly, PDX models in which fresh patient tumor tissues are directly transplanted into immunocompromised mice, represent a novel approach to identify personalized drug treatments. They are superior to standard cell line-derived xenografts because they maintain the histological, epigenetic and genetic characteristics across several passages, and includes the tumor microenvironment, which together creates a more realistic model of the pathophysiological conditions of the patient’s tumor.^257,258^ Indeed, copy number alterations found in PDX models of STS and bone sarcoma are also evident in sarcoma patients which suggest that these alterations are due to realistic tumor progression rather than model-specific artefacts.^259^ Furthermore, in vitro PDX cell lines and/or in vivo PDX mouse models enable researchers to perform high-throughput drug screens rapidly and inexpensively to design personalized treatments aimed at improving patient outcomes. Importantly, PDX models are highly predictive of clinical treatment response.^260^ This is evident in the response rates to standard chemotherapeutic and targeted therapies in PDX models correlating well with clinical outcome for a number of cancer patients including those with colorectal, pancreatic, non-small cell lung and breast cancer.^261–265^ Several studies have reported successful establishment of STS and bone sarcoma PDX models, with an overall engraftment success rate of 32–69% and successful recapitulation of the genetic and phenotypic characteristics of the original tumor.^266–270^ Furthermore, a high-throughput drug screen revealed that the most commonly used chemotherapeutics, HDAC and proteasome inhibitors, were active against most sarcoma subtypes.^268^ All rhabdomyosarcoma PDX models were particularly sensitive to the WEE1 inhibitor AZD1775 (PubChem CID: 24856436) and AZD1775 combined with current standard of care drugs vincristine (VCR) (PubChem CID: 5978) and irinotecan (IRN) (PubChem CID: 60838) had a better response rate in ERMS and ARMS PDX models compared to AZD1775, VCR and IRN alone. This demonstrates the advantage of using PDX models for personalized sarcoma therapy and justifies the use of a combination of the three drugs for future clinical trials. A summary of current personalized sarcoma treatment options is illustrated in Fig. 8.

## Conclusions

Despite the identification of oncogenic factors and pathways that drive sarcomagenesis and the development of therapies to target some of them, the treatment of sarcomas still poses a huge therapeutic challenge. This is due to the vast array of sarcoma subtypes, their intrinsic heterogeneity, alterations in many signaling pathways and variability in response to treatment. Not surprisingly, monotherapy has not been effective in the treatment of sarcomas, and combination therapies that target multiple oncogenic pathways while minimizing drug toxicity should be considered in future. It is anticipated that this will be achieved by unravelling the molecular mechanisms of each sarcoma subtype and applying personalized medicine principles to treat these highly aggressive and often drug resistant cancers.
